# Optimization of row and hill spacing patterns improved rice population structure and increased rice yield

**DOI:** 10.3389/fpls.2025.1570845

**Published:** 2025-05-26

**Authors:** Liqiang Dong, Tiexin Yang, Liang Ma, Yuedong Li

**Affiliations:** Liaoning Rice Research Institute, Liaoning Academy of Agricultural Sciences, Shenyang, Liaoning, China

**Keywords:** rice, row and hill spacing patterns, population structure, rice yield, wide narrow row densification mode

## Abstract

**Objective:**

The objective of this study was to investigate the effects of row and hill spacing patterns on rice populations during mechanized production, material accumulation and transport, and photosynthetic characteristics and to explore the role of these factors in improving rice yield.

**Methods:**

An field experiment was conducted from 2022 to 2023 with Liaojing 419 as the test material under four planting modes: LFM: local farmer cultivation mode was used as a control; and CDM: conventional densification mode; NDM: narrow row densification mode; and WNDM: wide narrow row densification mode were used as the treatment modes. Field mechanized transplantation experiments were conducted to investigate the effects of plant pattern on the physiological and morphological characteristics of rice plant individuals and populations, and clarify the relationships of physiological and biochemical changes with row and hill spacing.

**Results:**

The result shows that WNDM presented the greatest yield advantage in the two years trial, with yield significantly greater than that of the local farmer mode and other densification modes, with an increase of more than 8% compared with LFM. The reciprocal second leaf yielded the highest values, at 27.33 μmol·m^-2^·s^-1^ and 27.13 μmol·m^-2^·s^-1^under the WNDM over two years, which were significantly higher than those of the other modes. The WNDM resulted in the greatest accumulation of biomass during the heading–maturity stage, with values of 6.23 t/ha and 6.07 t/ha, respectively. Compared with the LFM, the WNDM had higher biomass at maturity, at 23.64 t/ha and 23.75 t/ha for two years, an increase of 17.90% and 17.87%, respectively. The sugar spikelets ratio was highest under the WNDM, which was significantly greater than those in the CDM and NDM.

**Conclusions:**

The wide narrow row densification mode of mechanized transplanting not only improved yield but also effectively optimized the population spatial distribution, improved resource utilization efficiency, and presented high production adaptability and promotion potential.

**Significance:**

This study provides theoretical and practical references for improving rice production efficiency and promoting high-quality mechanized processes in Northeast China, which is highly important for achieving sustainable development in modern agriculture.

## 1 Introduction

Rice is the second largest food crop in the world, supporting over half of the world’s population (Zheng et al., 2021; Gao et al., 2024). More than 90% of the energy required for crop yield formation comes from photosynthesis, whereas the average light energy conversion efficiency during the growth period is only 0.5%. Improving the light energy conversion efficiency of rice is a good potential method for increasing rice yield. As a major rice-consuming country, improvement in rice productivity and sustained increases in rice output are necessary for maintaining food security in China (Foley et al., 2011; Li et al., 2023; Deng et al., 2023).

Employing an appropriate crop planting pattern is important for establishing a reasonable population structure and improving light energy efficiency, which plays a crucial role in achieving relatively high yields (Dong et al., 2023; Xiong et al., 2021). The use of an appropriate row spacing can improve rice yield by optimizing the cultivation mode, by optimizing plant morphology by altering the planting pattern, by adjusting the plant row combinations to establish optimal population structures, and by adjusting the biomass to achieve root development (Peng et al., 2010, 2015; He et al., 2023). Li et al. (2015) studied the effect of plant spacing on canopy light interception capacity through manual transplanting methods and proposed that the light energy conversion rate improves at a reasonable density, and lodging is avoided due to increased light interception capacity, which is an effective way to increase crop yield. Ruan et al. (2022) suggested that in the context of climate change, to promote the sustainable development of rice production, the transplanting density of rice can be appropriately increased to improve the productive panicles number and fully realize the effective utilization of environmental resources to achieve good rice yield. He et al. (2023) studied the root characteristics and yield of rice under different planting modes and reported that an excessive number of tillers in the population resulted in limited individual space occupation, intensifying competition for resources for root growth and hindering the formation of high yields. Previous studies have explored row spacing effects on rice yield, however, two critical knowledge gaps remain in mechanized transplanting (Li et al., 2023; Dong et al., 2023). Most densification strategies prioritize yield gains at the expense of grain quality, yet no study has systematically quantified how spatial configurations modulate the source-sink-flow balance to achieve yield in japonica rice systems (Li et al., 2015: Wang et al., 2024). Existing research predominantly focuses on uniform row spacing adjustments, neglecting the synergistic potential of heterogeneous row configurations of alternating wide and narrow rows to reconcile light competition and mechanization constraints (Xiong et al., 2021; Dong et al., 2023; Cheng et al., 2024). Therefore, optimizing the plant spacing plays an important role in the formation of rice yield.

Liaoning, as the main planting and production base for japonica rice in northern China, has a relatively high yield of rice in the country (Li et al., 2023; Deng et al., 2023). However, continuous production over many years has led to significant differences in plant spacing, high spatiotemporal variation in plants, multiple tradeoffs between individuals and populations, and poor coordination between material accumulation and transport in this region (Peng et al., 2010, 2015). Issues related to rice yield and resource allocation and utilization in the region are becoming increasingly prominent, and a further understanding of the mechanisms by which high-efficiency planting results in changes in plant growth and morphology is urgently needed. However, research into the large-scale mechanized production of rice under different plant patterns is still in the exploratory stages (Xiong et al., 2022; Jia et al., 2023; Deng et al., 2024).

Therefore, in this study, a novel wide narrow row densification mode (WNDM) system that integrates spatial heterogeneity (row and hill spacing of 36 + 14 cm × 16 cm rows), narrow row densification mode (row and hill spacing of 25 cm × 17 cm), conventional densification mode (row and hill spacing of 30 cm × 14 cm) were selected, local farmer cultivation mode (row and hill spacing of 30 cm × 18 cm) was used as a control. The rice yield formation, leaf photosynthetic characteristics, biomass accumulation, photosynthate transport, and nonstructural carbohydrate characteristics under different modes, the rice yield formation trends under different hill and row spacing were analyzed, and a high-efficiency configuration for rice production in the local area was explored. The aim of the study was to provide a basis for high-yield mechanized cultivation measures for rice and reference for ensuring the sustainability of regional rice production.

## 2 Materials and methods

### 2.1 Experimental design

The rice (Oryza sativa L.) japonica cultivar ‘Liaojing401’ was used in this study. The tested cultivar belongs to a variety with a balance between panicles and grains that is currently the main cultivated variety in the central rice region of Liaoning Province (bred and provided by the Liaoning Rice Research Institute). It is a mid- to late-maturing variety with good balance between panicles and grains, with a potential yield of 13500 kg/hm^2^ in Liaoning Province. The number of grains per panicle is 128.7, and the thousand-grain weight is 24.5 g.

**Figure 1:**
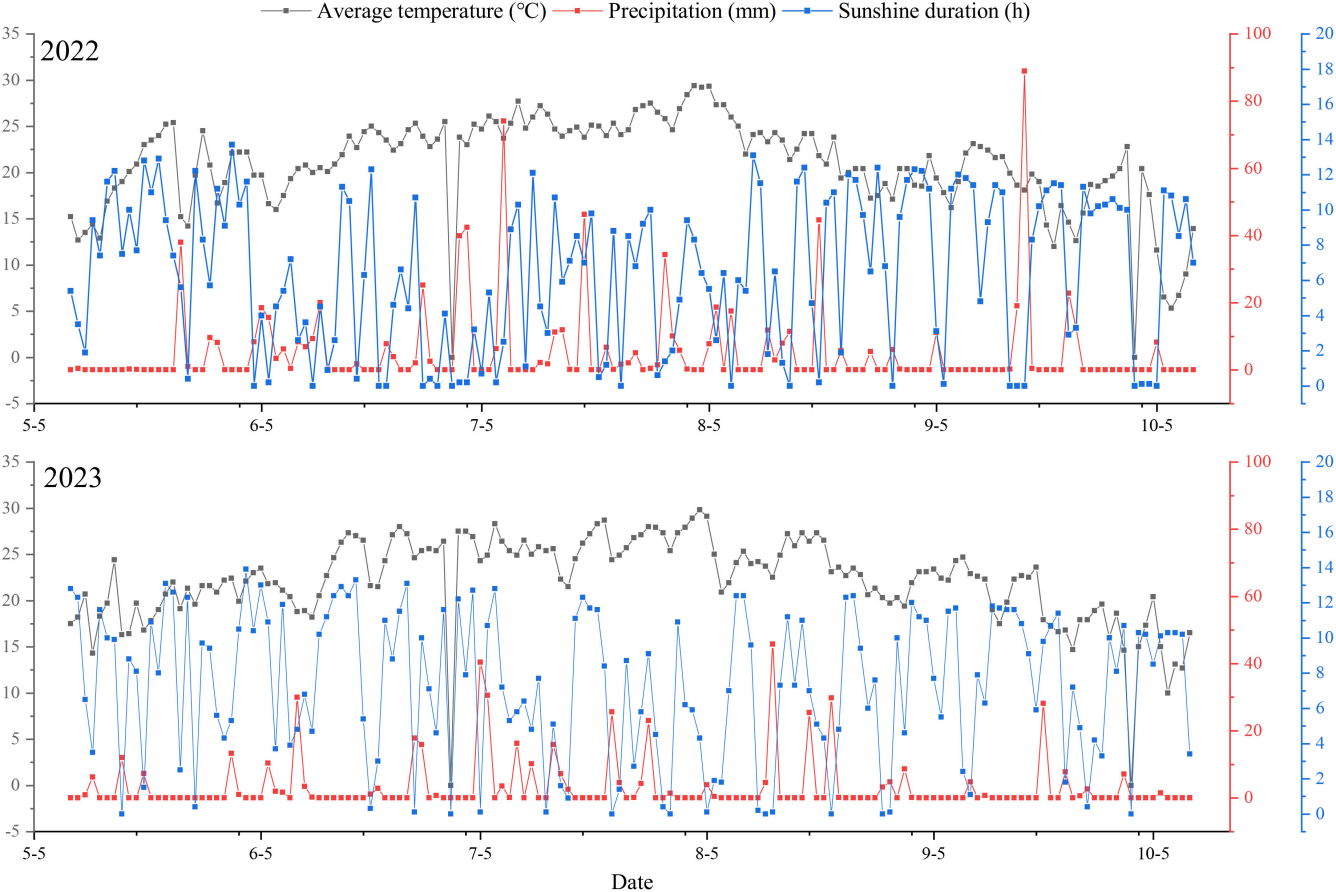
Meteorological data of rice growth period in the experimental site.

The field experiment was conducted at the experimental base of the Liaoning Rice Research Institute in Liutiaozhai town, Dengta city, Liaoning Province (E: 123.10°, N: 41.30°) of the Northeast China Plain, which has a warm temperate monsoon climate, the seasonal average annual precipitation and average maximum temperature were 694.5 mm and 20.6°C, respectively. The temperature, precipitation, and sunshine characteristics in this region are shown in **Figure 1**. As a rice-growing area in the central plain of Liaoning Province, the rice planting area and average yield in the region are relatively high.

The soil pH in the experimental area was 6.1, the organic matter content was 2.61%, the total nitrogen content was 1.44 g/kg, the total phosphorus content was 0.39 g/kg, and the total potassium content was 17.1 g/kg. The contents of available nitrogen, available phosphorus, and available potassium were 88.6 mg/kg, 48.1 mg/kg, and 85.4 mg/kg, respectively. In this study, the application rate of pure N (slow-release urea) was 300.0 kg/ha (base fertilizer: tiller fertilizer: panicle fertilizer = 6:3:1); the application rate of pure P (diammonium phosphate) was 69.0 kg/ha (base fertilizer); and the application rate of pure K (potassium chloride) was 75.0 kg/ha (base fertilizer: panicle fertilizer = 1:1).

In the field experiment, sowing was conducted on April 13 and April 14, 2022 and 2023; transplanting was performed on May 15 and May 16; and mechanized harvesting was carried out on October 5 and October 7 of the two years. The following four planting modes were used for treatment: LFM: Local farmer cultivation mode (row and hill spacing of 30 cm × 18 cm); CDM: conventional densification mode (row and hill spacing of 30 cm × 14 cm); NDM: narrow row densification mode (row and hill spacing of 25 cm × 17 cm); WNDM: wide narrow row densification mode (row and hill spacing of 36 + 14 cm × 16 cm). The transplanter was configured to alternate wide rows (36 cm) and narrow rows (14 cm) through a dual-row gearbox adjustment, while maintaining a fixed hill spacing of 16 cm. The seedling density was controlled by adjusting the rotating seedling tray frequency to 120 hills per minute. For the narrow row densification mode (25×16 cm), standard 25 cm row spacing plates were installed. All treatments equipped with an intelligent navigation system (Shanghai Lianshi Navigation Technology Co., Ltd.) was adopted, and 3~5 seedlings were planted each time. Field was conducted using a laser-guided system to ensure row alignment and spacing consistency across plots.

The mechanized transplanting area of the four modes was not less than 6670 m^2^; that is, the transplanting area was not less than 200 rows, and each row was not less than 100 m. After transplanting, three 500 m^2^ experimental investigation areas were separately divided into random groups for each mode as sampling areas for the measurement of each indicator. In the experimental area, well-water irrigation was used, the water management principle was “shallow water for live plants, drying (moistening) to promote tillering, irrigating with running water, and alternating dry and wet,” and field management methods such as transplanting seedlings with pesticides, biological traps, key growth period pesticide application for fly prevention, and anti-stress and disaster biological immune agent spraying were employed (Dong et al., 2021).

### 2.2 Index determination

#### 2.2.1 Growth dynamics

Stem and tiller dynamics: A stem and tiller count survey was conducted every 7 days from the time of rice transplanting to the full heading stage, and the number of stems and spikes during the transplanting, jointing, heading, full heading, and maturity stages was recorded.

#### 2.2.2 Rice yield and its components

Rice yield: After the maturity period, a Kubota 988 harvester (Kubota, Suzhou, China) was used for mechanized harvesting. After removing missing yields from the sample used, the yield was calculated on the basis of a moisture content of 14.5%.

Rice yield components: Before mechanized harvesting,10 plants with consistent growth were selected from each plot for indoor seed testing. The productive panicles per unit area, full grains number per panicle, seed setting rate, thousand seeds weight, etc., were measured.

#### 2.2.3 Leaf area index and dry matter accumulation

At the jointing stage, heading stage, and maturity stage, 4 representative plants were selected from each plot on the basis of the average tiller number, and the leaf area index was determined using the leaf area - weight ratio method (Wang et al., 2012). After the stems, leaves, and panicles were separated, the plants were blanched at 105°C for 30 min, dried at 80°C until a constant weight was reached, and then weighed (Dong et al., 2016). The content of nonstructural carbohydrates (NSCs) in the stem sheath during the heading and maturity stages was determined using a method described by Ren et al. (2022).

#### 2.2.4 Determination of photosynthesis-related indicators

At 10 days after heading stage, around August 10th, it is the period with the strongest temperature and sunlight of the season, 10 plants with consistent growth were selected from each plot. On sunny days from 9:00 to 10:00 in the morning, an LI-6400 portable photosynthesis meter (Lincoln, Nebraska, USA) was used to measure the net photosynthetic rate of flag leaves and reciprocal second leaves.

### 2.3 Data and computation

Calculate the tillers, stems, panicles and plant biomass parameter according to the following formula (Equations 1-8):


(1) $$\begin{array}{c} \text{T Total spikelets number }\left(/{\text{m}}^{2}\right)=\text{Productive panicles number }\left(/{\text{m}}^{2}\right)\\ \times \text{Full grains number}÷\text{Seed setting rate }\left(\%\right)\times 100 \end{array}$$

(2) $$\begin{array}{c} \text{Productive panicles rate}=\\ \text{Maturity panicles number}/\text{Maximum tillers number}\times 100 \end{array}$$

(3) $$\begin{array}{c} \text{Tiller capability}=\\ \text{Transplanting seedlings number}/\text{Maximum tillers number} \end{array}$$

(4) $$\begin{array}{c} \text{Leaf area index}\left(\text{LAI}\right)=\\ \text{Green leaf areas per unit areas}\times \text{Transplanting areas} \end{array}$$

(5) $$\text{Crop growth rate}\;(\text{g}\cdot {\text{m}}^{–2}\cdot {\text{d}}^{–1})=\left({\text{W}}_{2}–{\text{W}}_{l}\right)/\left({\text{t}}_{2}–{\text{t}}_{1}\right)$$

(6) $$\text{Leaf area attenuation rate }\left(\text{LAI}\cdot {\text{d}}^{–1}\right)=\left({\text{LAI}}_{2}–{\text{LAI}}_{1}\right)/\left({\text{t}}_{2}–{\text{t}}_{1}\right)$$

(7) $$\text{Leaf area daily }\left({\text{m}}^{2}\cdot \text{d}\right)=1/2\left({\text{L}}_{1}+{\text{L}}_{2}\right)\times \left({\text{t}}_{2}–{\text{t}}_{1}\right)$$

(8) $$\begin{array}{c} \text{Net assimilation rate},\;\left(\text{g}\cdot {\text{m}}^{–2}\cdot {\text{d}}^{–1}\right)\;=\left[\left({\text{ln LAI}}_{2}–{\text{ln LAI}}_{1}\right)/\left({\text{LAI}}_{2}–{\text{LA}}_{I1}\right)\right]\\ \;\times \left[\left({\text{W}}_{2}–{\text{W}}_{1}\right)/\left({\text{t}}_{2}–{\text{t}}_{1}\right)\right] \end{array}$$

In the formula, W_1_ and W_2_ represent the dry matter weight measured twice before and after, t_1_ and t_2_ represent the time measured twice before and after, and LAI_1_ and LAI_2_ represent the leaf area index measured twice before and after, L_1_ and L_2_ represent the leaf area measured twice before and after.

Calculate the NSC transport rate after rice heading, sugar spikeletes ratio at heading stage, pre-anthesis material transport amount and transport rate according to the following formula (Equations 9, 10):


(9) $$\text{NSC transport rate}\left(\%\right)=\left[\text{NSC accumulation in stem sheath at heading stage }\left(\text{g}/{\text{m}}^{2}\right)-\text{NSC accumulation in mature stem sheath }\left(\text{g}/{\text{m}}^{2}\right)\right]/\text{NSC content in stem sheath at heading stage }\left(\text{g}/{\text{m}}^{2}\right)\times 100$$

(10) $$\text{Sugar spikelet ratio }\left(\text{mg}/\text{spikelet}\right)=\text{NSC accumulation in stems and leaf sheaths at heading stage}\left(\text{g}/{\text{m}}^{2}\right)/\text{total spikeletesnumber}\left(\text{spikelet}/{\text{m}}^{2}\right)$$

### 2.4 Statistical analysis

Excel (2020, Microsoft, Redmond, WA, USA) was used for data organization, SPSS (22.0; SPSS Inc., Chicago, IL, USA) was used for the analysis of variance (ANOVA) to evaluate significant differences, and Origin (Origin Lab, Hampton, MA, USA) was used for data plotting. All data are expressed as the mean ± standard deviation with three replicates. Multiple comparisons of means were based on Fisher’s least significant difference (LSD) test at a 5% significance level unless stated otherwise. A structural equation model was used to calculate the path coefficients between yield, transplanting quality, and physiological and ecological indicators by using Smart PLS4.

## 3 Result

### 3.1 Actual yield

**Figure 2:**
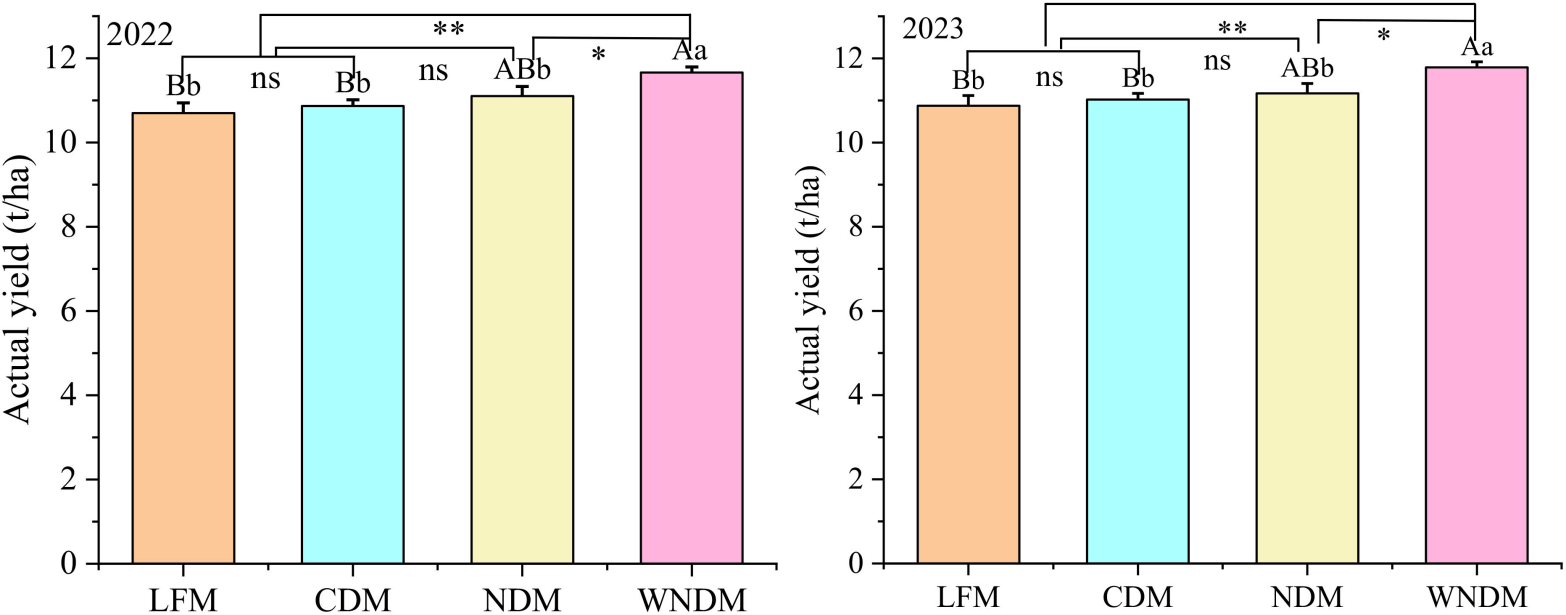
Effects of hill and row distances on actual rice yield. A one-way analysis of variance (ANOVA) followed by least significant difference (LSD) multiple range test was per-formed to evaluate the statistical differences among the treatments per each parameter measured. Different uppercase letters and lowercase letters indicate significant difference at *P< 0.05* and *P< 0.01* probability level in a year, respectively. * indicates the *P< 0.05*, ** indicates the *P< 0.01*, ns indicates no significant.

Actual harvest quality of grains is a key indicator used to measure the production efficiency of rice. According to the analysis of the actual yields measured in the field over two years under four different modes (**Figure 2**), the yield performance trends in 2022 and 2023 were consistent, with each cultivation mode showing slightly higher yield in 2023 than in 2022. The yield of the LFM was the lowest in the two years, and the yields of three densification modes were greater than those of LFM. The WNDM had the highest yield, which was significantly (P< 0.05) greater than that of NDM and extremely significantly (P< 0.01) greater than those of LFM and CDM. In 2022 and 2023, these values were 11.66 t/ha and 11.79 t/ha, respectively, which were 8.98% and 8.43% greater than those of the lowest yielding mode, the LFM. There was no significant (P > 0.05) difference in yield among the LFM, CDM, and DNM.

### 3.2 Yield components

Rice yield composition determines the theoretical yield, which indirectly reflects the actual yield. According to the analysis of the yield composition factors under the four modes (**Table 1**), there were significant (P< 0.05) differences in the productive panicles number between years and modes, with that under the LFM being significantly (P< 0.05) lower than that under the other treatments. The NDM resulted in the highest number of productive panicles, with the two years of 433.72 and 441.70/m^2^, increasing by 78.14 and 75.79/m^2^ compared with the LFM. The WNDM resulted in the second highest number of productive panicles, with the two of 431.36 and 435.47/m^2^, increasing by 73.70 and 67.48/m^2^ compared with LFM. The full grains number under the CDM, NDM and WNDM was significantly (P< 0.05) lower than the LFM. The seed setting rate of the LFM was the highest, and significantly (P< 0.05) greater than those of the CDM and NDM. There was no significant (P> 0.05) difference in thousand seeds weight among the treatments. The total spikelets number under the LFM was significantly (P< 0.05) lower than that under the other treatments, indicating that densification modes increased storage capacity compared with the LFM, providing a basis for yield formation.

**Table 1:** Effects of hill and row distances on rice yield components.

Year	Mode	Productive panicles number (/m^2)^	Full grains number	Seed setting rate (%)	Thousand seeds weight (g)	Total spikelets number (10^3^/m^2^)
2022	LFM	355.58 ± 1.92Bc	124.69 ± 2.63Aa	91.23 ± 1.56Aa	24.99 ± 0.57Aa	48.59 ± 1.13Bb
	CDM	417.18 ± 5.69Ab	111.90 ± 1.32Bb	85.88 ± 0.69ABbc	24.27 ± 0.33Aa	54.36 ± 0.73Aa
	NDM	433.72 ± 1.07Aa	109.17 ± 2.45Bb	82.88 ± 2.17Bc	24.58 ± 0.51Aa	57.14 ± 1.20Aa
	WNDM	431.36 ± 4.84Aa	115.24 ± 1.50ABb	87.61 ± 1.47ABab	24.62 ± 0.28Aa	56.75 ± 0.66Aa
2023	LFM	368.00 ± 0.43Cc	122.60 ± 2.59Aa	93.21 ± 1.60Aa	25.14 ± 0.57Aa	48.41 ± 1.06Bb
	CDM	424.50 ± 2.00Bb	108.95 ± 1.29Bbc	86.39 ± 0.69Bbc	24.97 ± 0.34Aa	53.54 ± 0.72Aa
	NDM	441.70 ± 2.11Aa	106.80 ± 2.40Bc	83.84 ± 2.20Bc	24.84 ± 0.51Aa	56.27 ± 1.15Aa
	WNDM	435.47 ± 3.68ABa	113.85 ± 1.47ABb	88.11 ± 1.48ABb	24.92 ± 0.28Aa	56.27 ± 0.63Aa
	Y	**	*	ns	ns	ns
	M	**	**	ns	ns	**
	Y×M	ns	ns	ns	ns	ns

Data in the table are means ± standard deviation, with three replicates. A one-way analysis of variance (ANOVA) followed by least significant difference (LSD) multiple range test was per-formed to evaluate the statistical differences among the treatments per each parameter measured. Different uppercase letters and lowercase letters indicate significant difference at *P< 0.05* and *P< 0.01* probability level within a column in a year, respectively. A two-way ANOVA was used to test the effects of mode, year, and their interactions. Y·indicates the year, M·indicates the mode,·Y × M·indicates the interaction between year and mode. * indicates the *P< 0.05*, ** indicates the *P< 0.01*, ns indicates no significant.

### 3.3 Tiller formation

Tillering and stem formation determine the number of productive panicles during the maturity period. The analysis of dynamic changes in stem and panicle number under the four different modes (**Figure 2**) revealed that the trend in stem and tiller number changes was consistent across years, with that in 2023 being slightly greater than that in 2022, showing a rapid increase with the growing period, reaching its highest point at the heading stage, then decreasing at the full heading stage, and finally stabilizing at the maturity stage. Compared with the other treatments, the LFM resulted in significantly (P< 0.05) lower values at all growth stages, with the NDM showing the highest value except at the heading stage in 2022.

**Figure 3:**
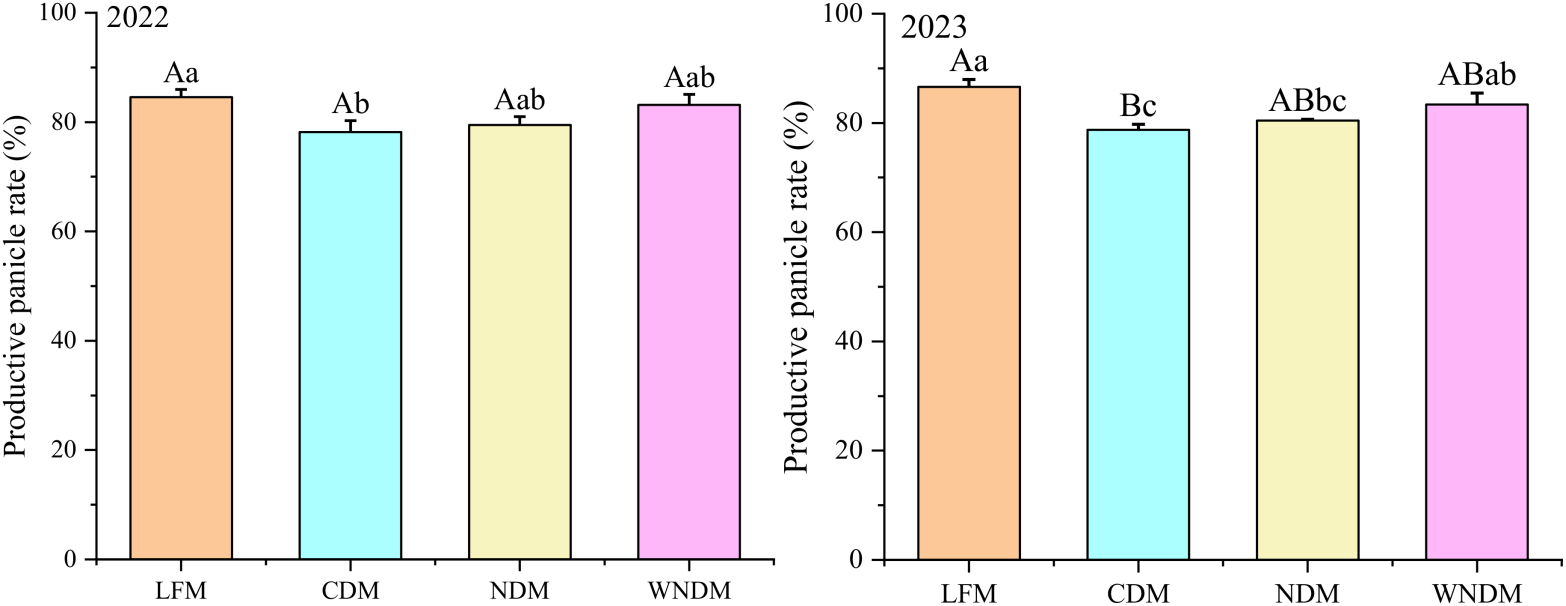
Productive panicles rate. A one-way analysis of variance (ANOVA) followed by least significant difference (LSD) multiple range test was per-formed to evaluate the statistical differences among the treatments per each parameter measured. Different uppercase letters and lowercase letters indicate significant difference at P< 0.05 and P< 0.01 probability level in a year, respectively.

**Figure 4:**
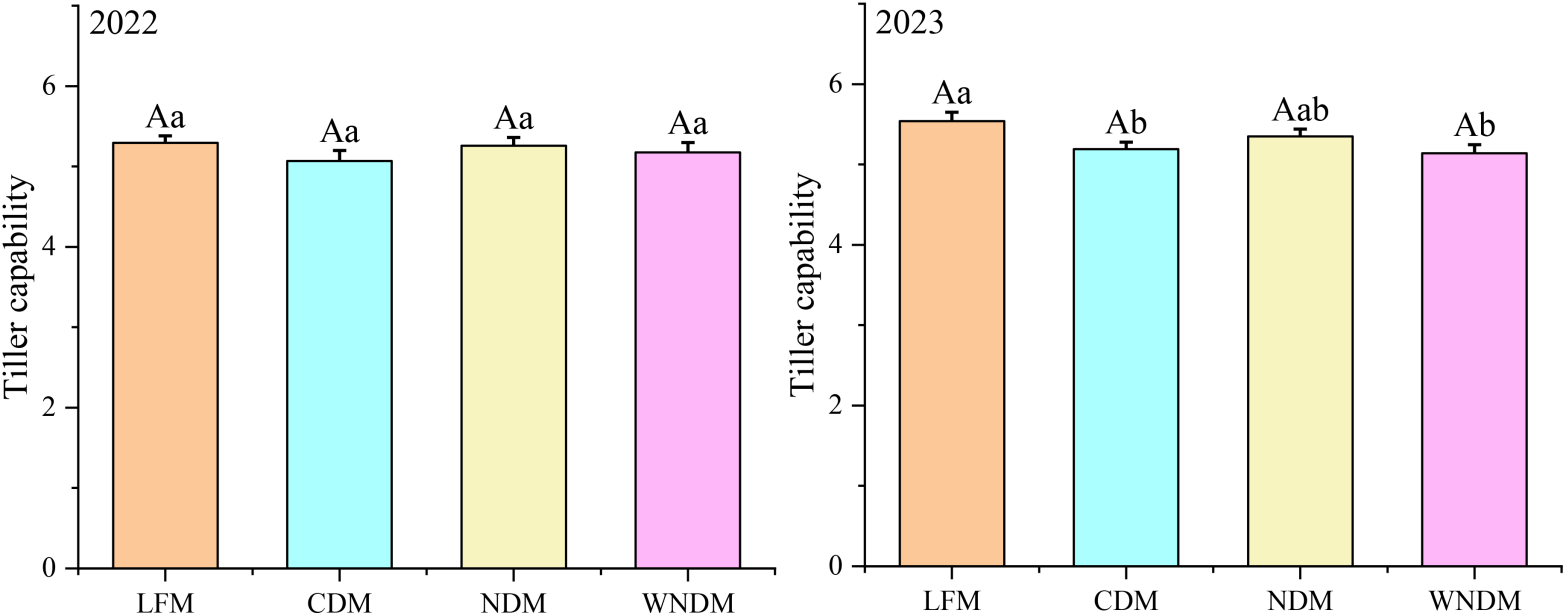
Tiller capacity. A one-way analysis of variance (ANOVA) followed by least significant difference (LSD) multiple range test was per-formed to evaluate the statistical differences among the treatments per each parameter measured. Different uppercase letters and lowercase letters indicate significant difference at *P< 0.05* and *P< 0.01* probability level in a year, respectively.

**Figure 5:**
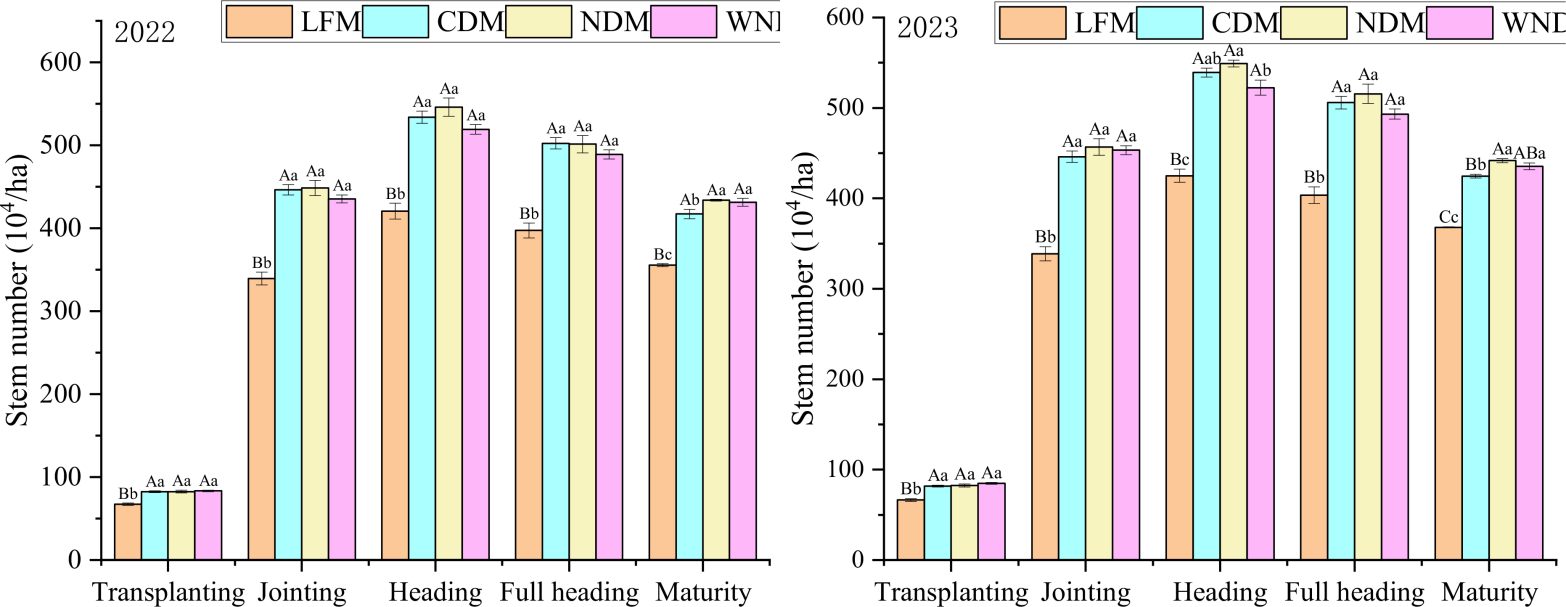
Tiller formation. A one-way analysis of variance (ANOVA) followed by least significant difference (LSD) multiple range test was per-formed to evaluate the statistical differences among the treatments per each parameter measured. Different uppercase letters and lowercase letters indicate significant difference at *P< 0.05* and *P< 0.01* probability level in a year, respectively.

The maximum number of tillers is based on basic seedling number and tillering capability (**Figures 3**, **4**). The LFM resulted in the greatest tillering capability in the two years, with values of 5.30 and 5.54, respectively. In 2023, the tillering capability under the LFM was significantly higher than the CDM and WNDM; that of the NDM was the second highest, with values of 5.26 and 5.35 in the two years. The productive panicle rate is the most important indicator during the maturity stage, reflecting the population structure (**Figure 5**). Over the two years, the LFM had the highest values of the productive panicle rate, at 84.56% and 86.60%. The productive panicles rates of the WNDM at two years were 83.11% and 83.37%, respectively, which were significantly (P< 0.05) higher than those of the CDM and NDM in 2023, indicating that this mode has advantages over other densification modes in terms of the productive panicles rate.

### 3.4 Biomass

**Figure 6:**
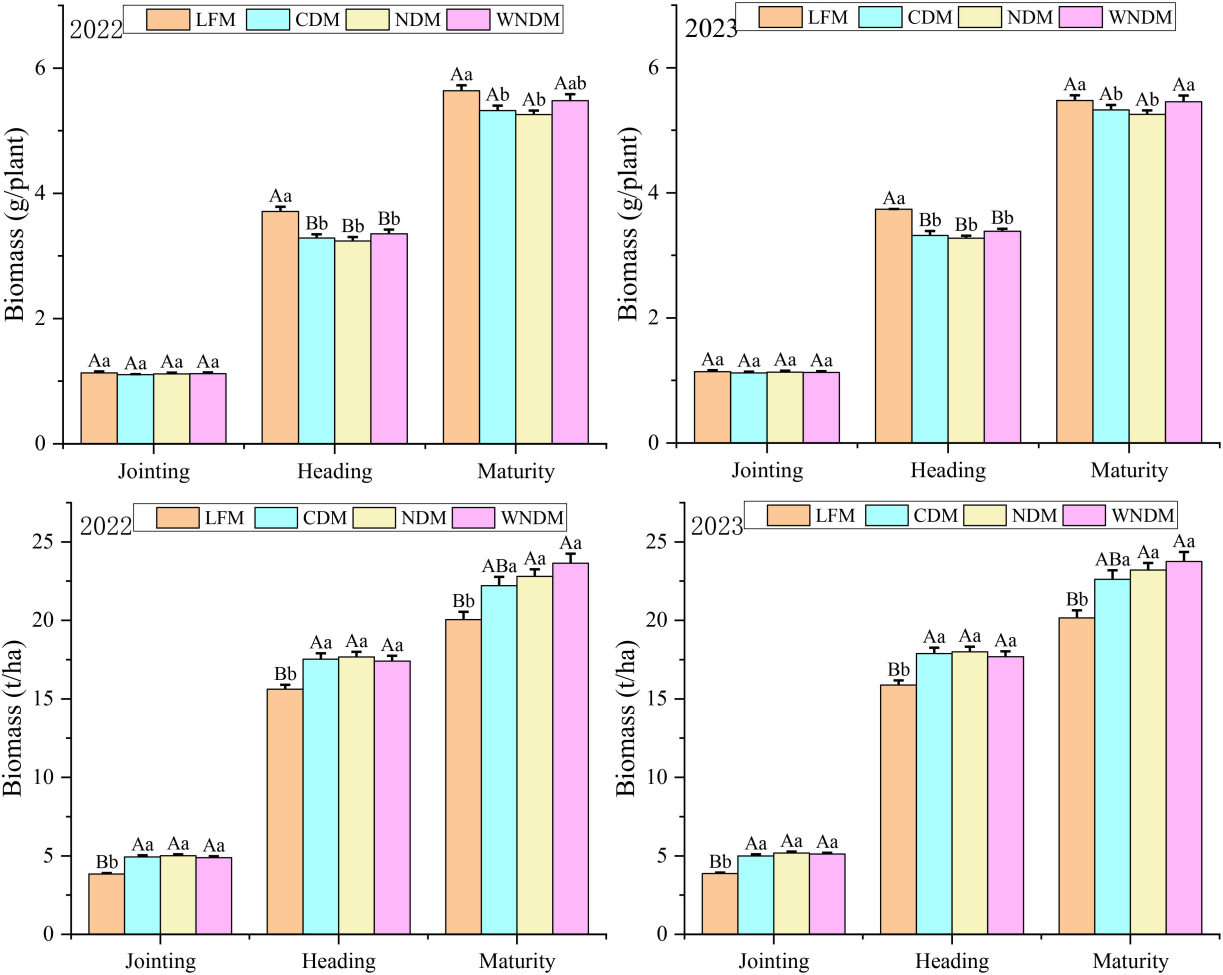
Biomass accumulation. A one-way analysis of variance (ANOVA) followed by least significant difference (LSD) multiple range test was per-formed to evaluate the statistical differences among the treatments per each parameter measured. Different uppercase letters and lowercase letters indicate significant difference at *P< 0.05* and *P< 0.01* probability level in a year, respectively.

Biomass is an important indicator for measuring the material accumulation process of a single stem and population of plants and is the material basis for rice yield. It is measured, calculated, and analyzed for individuals and populations of plants (**Figure 6**). There was no significant (P> 0.05) difference in the accumulation of dry matter in single stems among the treatments during the jointing stage, but that for the LFM at the heading stage was significantly (P< 0.05) greater than that of the other modes. At maturity, the LFM still exhibited significantly (P< 0.05) greater maximum accumulation in a single stem than that under the CDM and NDM. The accumulation of dry matter in the population was the lowest under the LFM, which was significantly (P< 0.05) lower than that in the other densification modes, because the lowest number of tillers were obtained under this mode. The NDM at the heading stage had the highest value, with values of 17.7 t/ha and 18.0 t/ha for the 2 years. Compared with the LFM, the WNDM had the highest biomass during the mature stage, with 23.64 t/ha and 23.75 t/ha at two years, respectively, an increase of 17.90% and 17.87%, indicating that the WNDM is beneficial for the accumulation of biomass.

### 3.5 Population growth rate

The accumulation of biomass in different phases reflects the growth rate and quality of a rice population, and analyses are shown in **Table 2**. The accumulation of biomass during the jointing to heading stage was significantly (P< 0.05) greater with the CDM and NDM than with the LFM. The WNDM resulted in the greatest biomass accumulation during the heading–maturity stage, at 6.23 t/ha and 6.07 t/ha in the two years, which had a value significantly (P< 0.05) greater than those of the other modes, except under the NDM in 2023. The growth rate of the group followed the same pattern as that of dry matter accumulation during different stages, with the growth rate of the group during the jointing stage being slightly greater in 2023 than in 2022. The accumulation of substances during the jointing to heading stage accounted for the greatest proportion of total biomass under the LFM, at 58.72% and 59.62%, respectively, which was significantly (P< 0.05) greater than that of the other modes. The WNDM had the greatest proportion of total biomass during the heading to maturity stage, at 26.36% and 25.53% in the two years, indicating that the WNDM was beneficial for material transport during the heading to maturity stage.

**Table 2:** Population growth rate.

Year	Mode	Phase accumulation (t/ha)		Population growth rate (g/m^2^/d)		Ratio to Total (%)	
		Jointing–heading	Heading–maturity	Jointing–heading	Heading–maturity	Jointing–heading	Heading–maturity
2022	LFM	11.77 ± 0.36Ab	4.44 ± 0.19Bc	56.07 ± 1.73Ab	7.40 ± 0.32Bc	58.72 ± 0.17Aa	22.15 ± 0.23Bbc
	CDM	12.60 ± 0.40Aa	4.67 ± 0.20Bbc	60.01 ± 1.89Aa	7.79 ± 0.33Bbc	56.75 ± 0.13Bb	21.04 ± 0.18Bc
	NDM	12.66 ± 0.10Aa	5.13 ± 0.13Bb	60.30 ± 0.47Aa	8.54 ± 0.22Bb	55.53 ± 0.22Bc	22.48 ± 0.31Bb
	WNDM	12.53 ± 0.10Aab	6.23 ± 0.28Aa	59.65 ± 0.49Aab	10.39 ± 0.47Aa	52.98 ± 0.52Cd	26.36 ± 0.72Aa
2023	LFM	12.01 ± 0.37Ab	4.27 ± 0.19Bc	57.21 ± 1.76Ab	7.12 ± 0.31Bc	59.62 ± 0.18Aa	21.19 ± 0.23Bc
	CDM	12.90 ± 0.41Aa	4.72 ± 0.20Bbc	61.41 ± 1.93Aa	7.86 ± 0.33Bbc	57.04 ± 0.13Bb	20.87 ± 0.18Bc
	NDM	12.81 ± 0.10Aa	5.21 ± 0.13ABb	61.01 ± 0.47Aa	8.69 ± 0.22ABb	55.22 ± 0.22Cc	22.47 ± 0.31Bb
	WNDM	12.58 ± 0.10Aab	6.07 ± 0.28Aa	59.89 ± 0.49Aab	10.11 ± 0.47Aa	52.95 ± 0.51Dd	25.53 ± 0.72Aa
	Y	ns	ns	ns	ns	ns	*
	M	**	**	**	**	**	**
	Y×M	ns	ns	ns	ns	*	ns

Data in the table are means ± standard deviation, with three replicates. A one-way analysis of variance (ANOVA) followed by least significant difference (LSD) multiple range test was per-formed to evaluate the statistical differences among the treatments per each parameter measured. Different uppercase letters and lowercase letters indicate significant difference at *P< 0.05* and *P< 0.01* probability level within a column in a year, respectively. A two-way ANOVA was used to test the effects of mode, year, and their interactions. Y·indicates the year, M·indicates the mode,·Y × M·indicates the interaction between year and mode. * indicates the *P< 0.05*, ** indicates the *P< 0.01*, ns indicates no significant.

### 3.6 Leaf characteristics

Leaves are important sites for photosynthesis and sources of material accumulation in rice. Different methods of leaf trait determination and analysis were used (**Table 3**). The leaf area index (LAI) of the LFM in the jointing stage was significantly lower than the other modes. Except under the CDM in 2022, the leaf area index under the LFM was significantly (P< 0.05) lower than other modes in the heading stage, and significantly (P< 0.05) lower than that of the NDM and WNDM in the maturity stage. The WNDM resulted in the highest heading LAI in 2022, at 6.89, whereas the NDM had the highest heading LAI in 2023, at 7.03. The decay rate of leaf area was lower under the LFM, significantly (P< 0.05) lower than that under the densification modes, in 2023, indicating that the densification mode increased leaf aging. The photosynthetic potential during the jointing to heading stage and heading to maturity stage was highest under the NDM and was significantly (P< 0.05) greater than that under the LFM during the jointing to heading stage over the two years. In the heading to maturity stage, photosynthetic potential was highest in the NDM. The net assimilation rate (NAR) reflects the comprehensive efficiency with which crop photosynthetic products are assimilated for material accumulation. It is an important parameter for measuring crop growth performance and light energy utilization efficiency. During the jointing to heading stage, the LFM had a significantly (P< 0.05) greater effect on NAR than the other modes. During the heading to maturity stage, the WNDM had a significantly (P< 0.05) greater effect than the other modes, indicating that the WNDM resulted in greater material accumulation and transport in the later stages of growth.

**Table 3:** Leaf characteristics.

Year	Mode	Leaf area index			Decay rate of leaf area	Photosynthetic potential (m^2^·d)		Net assimilation rate (g·m^–2^·d^–1^)	
		Jointing	Heading	Maturity	(LAI·d^–1^)	Jointing–heading	Heading–maturity	Jointing–heading	Heading–maturity
2022	LFM	1.93 ± 0.06Bb	6.22 ± 0.23Ab	4.70 ± 0.02Ab	0.0253 ± 0.0035Ab	85.52 ± 2.31Bb	327.60 ± 6.31Ab	15.33 ± 0.04Aa	1.37 ± 0.01Bc
	CDM	2.45 ± 0.08Aa	6.64 ± 0.24Aab	4.77 ± 0.12Aab	0.0311 ± 0.0020Aab	95.4 ± 2.63ABa	342.21 ± 9.68Aab	14.31 ± 0.07Bb	1.38 ± 0.02Bbc
	NDM	2.51 ± 0.02Aa	6.87 ± 0.11Aa	5.01 ± 0.08Aa	0.0310 ± 0.0006Aab	98.51 ± 2.01Aa	356.35 ± 6.49Aa	13.92 ± 0.28Bb	1.45 ± 0.04Bb
	WNDM	2.46 ± 0.02Aa	6.89 ± 0.19Aa	4.89 ± 0.09Aab	0.0333 ± 0.0017Aa	98.12 ± 2.92Aa	353.36 ± 9.56Aab	13.88 ± 0.39Bb	1.78 ± 0.02Aa
2023	LFM	1.93 ± 0.06Bb	5.96 ± 0.22Bb	4.61 ± 0.12Ab	0.0225 ± 0.0017Bb	82.83 ± 2.24Bb	317.03 ± 8.75Bb	16.03 ± 0.06Aa	1.36 ± 0.05Bc
	CDM	2.47 ± 0.08Aa	6.76 ± 0.24ABa	4.89 ± 0.12Aab	0.0312 ± 0.0020Aa	96.89 ± 2.71Aa	349.55 ± 9.71ABa	14.44 ± 0.06Bb	1.36 ± 0.04Bc
	NDM	2.59 ± 0.02Aa	7.03 ± 0.11Aa	5.05 ± 0.14Aa	0.0331 ± 0.0004Aa	101.03 ± 2.02Aa	362.40 ± 8.56Aa	13.72 ± 0.27BCc	1.45 ± 0.03Bb
	WNDM	2.54 ± 0.02Aa	7.00 ± 0.20Aa	4.94 ± 0.09Aab	0.0342 ± 0.0017Aa	100.17 ± 2.93Aa	358.12 ± 9.90ABa	13.61 ± 0.38Cc	1.71 ± 0.02Aa
	Y	*	ns	ns	ns	ns	ns	ns	ns
	M	**	**	**	**	**	**	**	**
	Y×M	ns	ns	ns	ns	ns	ns	*	ns

Data in the table are means ± standard deviation, with three replicates. A one-way analysis of variance (ANOVA) followed by least significant difference (LSD) multiple range test was per-formed to evaluate the statistical differences among the treatments per each parameter measured. Different uppercase letters and lowercase letters indicate significant difference at *P< 0.05* and *P< 0.01* probability level within a column in a year, respectively. A two-way ANOVA was used to test the effects of mode, year, and their interactions. Y·indicates the year, M·indicates the mode,·Y × M·indicates the interaction between year and mode. * indicates the *P< 0.05*, ** indicates the *P< 0.01*, ns indicates no significant.

### 3.7 Photosynthetic parameters

Photosynthetic parameters reflect the photosynthetic capacity of leaves, and the determination and analysis of the photosynthetic traits of leaves 10 days after heading were performed in different modes (**Table 4**). The photosynthetic rate of the flag leaf was greater than that of the reciprocal second leaf, and the photosynthetic rate of the flag leaf was significantly (P< 0.05) greater under LFM than that in the other modes. The reciprocal second leaf under WNDM had highest values for the photosynthetic rate, at 27.33 μmol·m^-2^·s^-1^ and 27.13 μmol·m^-2^·s^-1^ over the two years, which were significantly (P< 0.05) greater than those of the other modes. The photosynthetic rate of the reciprocal second leaf in the WNDM decreased less than that in the flag leaf. In 2022, the stomatal conductance was highest in the flag leaf and the reciprocal second leaf under the LFM. In 2023, the stomatal conductance of the flag leaf was highest under the WNDM, and that of the reciprocal second leaf was the highest under the LFM. The intercellular CO_2_ concentration in flag leaves was highest with the CDM in 2022 and with the NDM in 2023. The intercellular CO_2_ concentration in reciprocal second leaf was the highest under the LFM in 2022, and it was the highest under the NDM in 2023. The transpiration rate of flag leaves was highest under the CDM in 2022 and was highest under the WNDM in 2023. The transpiration rate of the reciprocal second leaf was significantly lower under the NDM than under the other modes in 2022 and 2023.

**Table 4:** Photosynthetic parameters.

Year	Mode	Photosynthetic rate (μmol·m^-2^·s^-1^)		Stomatal conductance (mol·m^-2^·s^-1^)		Intercellular CO_2_ concentration (μmol·mol^-1^)		Transpiration rate (mmol·m^-2^·s^-1^)	
		Flag leaf	Reciprocal second leaf	Flag leaf	Reciprocal second leaf	Flag leaf	Reciprocal second leaf	Flag leaf	Reciprocal second leaf
2022	LFM	29.07 ± 0.12Aa	25.67 ± 0.50Ab	0.393 ± 0.001ABb	0.404 ± 0.007Aa	266.67 ± 0.58Bd	287.67 ± 4.16Aa	7.16 ± 0.01Bb	7.44 ± 0.36Aa
	CDM	27.23 ± 0.21BCc	25.30 ± 0.17Ab	0.403 ± 0.001Aa	0.396 ± 0.003Aab	281.67 ± 0.58Aa	287.33 ± 0.58Aab	7.49 ± 0.02Aa	7.01 ± 0.04Aa
	NDM	26.60 ± 0.20Cc	21.53 ± 0.91Bc	0.385 ± 0.004Bb	0.333 ± 0.004Bc	277.67 ± 2.08Ab	270.33 ± 3.79Abc	7.09 ± 0.08Bb	5.18 ± 0.04Bb
	WNDM	27.97 ± 0.42Bb	27.33 ± 0.25Aa	0.386 ± 0.003Bb	0.367 ± 0.028ABbc	270.33 ± 1.53Bc	270 ± 9.54Ac	7.05 ± 0.03Bb	6.92 ± 0.45Aa
2023	LFM	31.17 ± 0.40Aa	25.83 ± 0.67Ab	0.476 ± 0.001Aa	0.405 ± 0.010Aa	195.00 ± 1.73Bc	287.33 ± 4.93Aa	9.68 ± 0.01Aa	7.24 ± 0.24Aab
	CDM	26.53 ± 0.15BCc	25.83 ± 0.21Ab	0.386 ± 0.006Cc	0.401 ± 0.007Aa	280.00 ± 1.73Aa	286.67 ± 1.53Aa	7.23 ± 0.1Cc	7.38 ± 0.36Aa
	NDM	25.33 ± 0.29Cc	23.00 ± 0.26Bc	0.405 ± 0.002Bb	0.377 ± 0.001ABb	289.33 ± 1.53Aa	289.33 ± 1.53Aa	7.59 ± 0.03Bb	5.84 ± 0.02Bc
	WNDM	28.73 ± 1.20Bb	27.13 ± 0.06Aa	0.477 ± 0.002Aa	0.345 ± 0.008Bc	207.33 ± 6.51Bb	265 ± 2.65Bb	9.73 ± 0.03Aa	6.63 ± 0.12ABb
	Y	ns	*	**	ns	**	ns	**	ns
	M	**	**	**	**	**	**	**	**
	Y×M	**	*	**	*	**	*	**	*

Data in the table are means ± standard deviation, with three replicates. A one-way analysis of variance (ANOVA) followed by least significant difference (LSD) multiple range test was per-formed to evaluate the statistical differences among the treatments per each parameter measured. Different uppercase letters and lowercase letters indicate significant difference at *P< 0.05* and *P< 0.01* probability level within a column in a year, respectively. A two-way ANOVA was used to test the effects of mode, year, and their interactions. Y·indicates the year, M·indicates the mode,·Y × M·indicates the interaction between year and mode. * indicates the *P< 0.05*, ** indicates the *P< 0.01*, ns indicates no significant.

### 3.8 Stem sheath NSC transport rate and sugar spikeletes ratio at the heading stage

**Figure 7:**
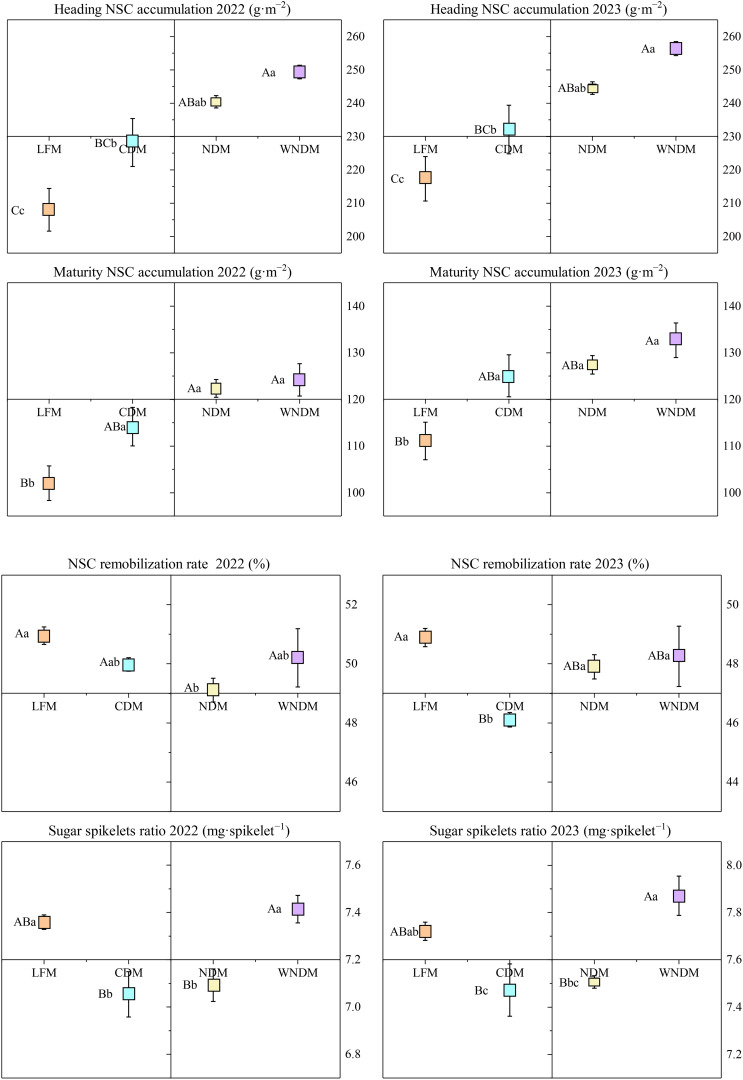
Nonstructural carbohydrate (NSC) content in stems and sugar spikelets ratio at heading to NSC remobilization. A one-way analysis of variance (ANOVA) followed by least significant difference (LSD) multiple range test was per-formed to evaluate the statistical differences among the treatments per each parameter measured. Different uppercase letters and lowercase letters indicate significant difference at *P< 0.05* and *P< 0.01* probability level within a column in a year, respectively.

The accumulation and transport of NSCs in the stem sheath can facilitate the “extraction” of grain sink from storage organs; therefore, the NSCs in stems were analyzed (**Figure 7**). NSC accumulation was lowest under the LFM and highest under the WNDM at the heading stage and maturity stage, respectively, and the difference between two modes was highly significant. NSC accumulation in the maturity stage was lower under the CDM than under the NDM and WNDM, and it was significantly (P< 0.05) higher in the densification modes than in the LFM, indicating that densification is beneficial for the accumulation of NSCs. The NSC transport rate was highest under the LFM, followed by the WNDM. The sugar spikelets ratio was highest under the WNDM, which was significantly (P< 0.05) greater than that in the CDM and NDM. These findings indicate that the WNDM resulted in a relatively high transport rate as determined by observations of high NSC accumulation, providing abundant carbohydrates for grain material accumulation.

### 3.9 Correlations between agronomic traits and yield in rice

**Figure 8:**
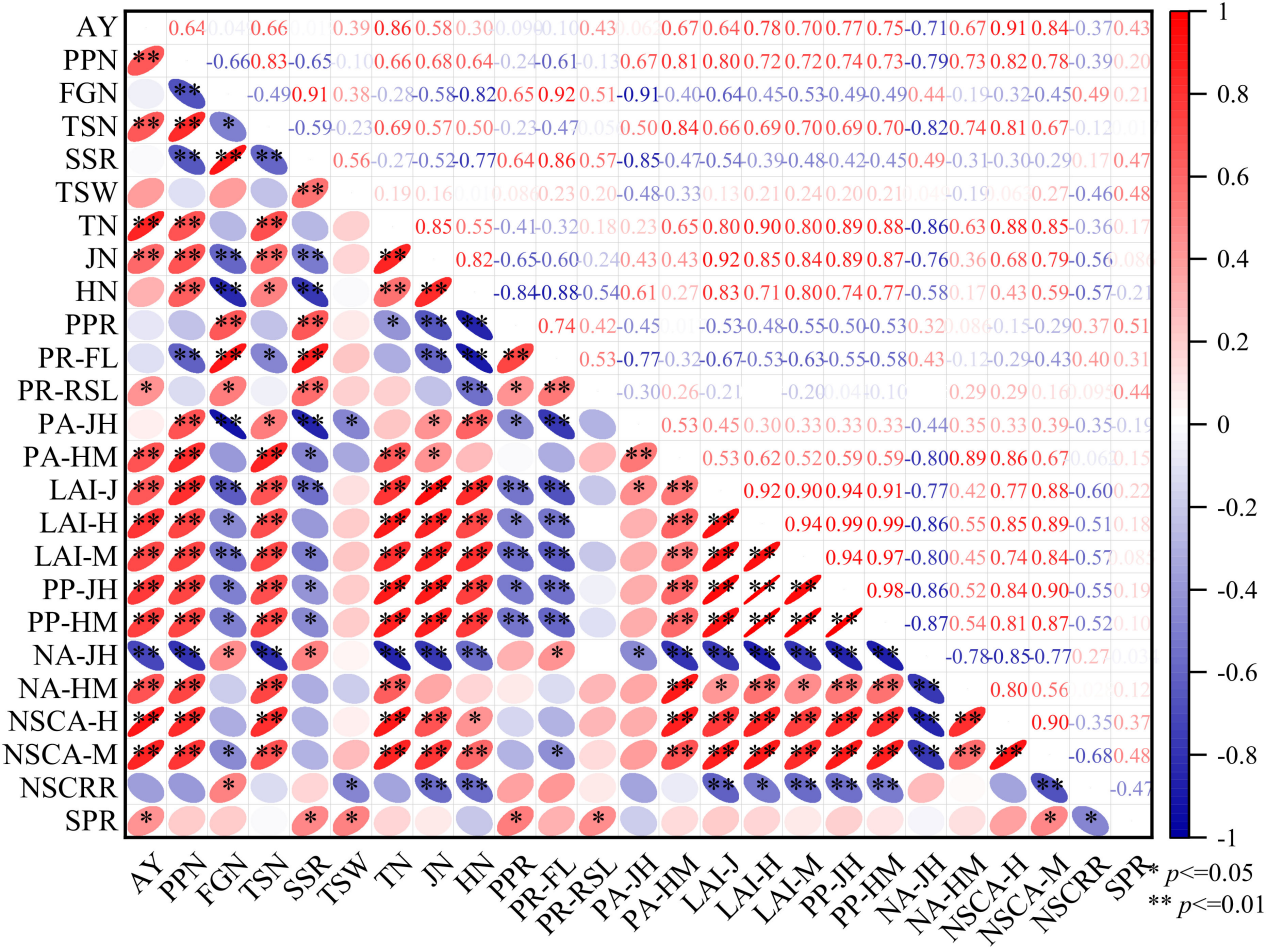
Correlations between agronomic traits. AY is actual yield, PPN is productive panicles number, FGN is full grains number, TSN is total spikelets number, SSR is seed setting rate, TSW is thousand seeds weight, TN is stems number of transplanting stage, JN is stems number of jointing stage, HN is stems number of heading stage, PPR is productive panicles rate, PR-FL is photosynthetic rate of flag leaf, PR-RSL is photosynthetic rate of reciprocal second leaf, PA-JH is phase accumulation of jointing to heading, PA-HM is phase accumulation of heading to maturity, LAI-J is leaf area index of jointing, LAI-H is leaf area index of heading, LAI-J is leaf area index of maturity, PP-JH is photosynthetic potential of jointing to heading, PP-HM is photosynthetic potential of heading to maturity, NA-JH is net assimilation of jointing to heading, NA-HM is net assimilation of heading to maturity, NSCA-H is NSC accumulation of heading, NSCA-M is NSC accumulation of maturity. * indicates *P< 0.05*, ** indicates *P< =0.01*.

Compared with the LFM, the densification modes can increase the productive panicles number, promote the transport of assimilates to grains, and increase the full grains number while ensuring a greater productive panicles number, thereby improving rice yield (**Figure 2**; **Table 1**). Correlations between agronomic traits and yield in rice were conducted (**Figure 8**, Correlation type: Sperman, Exclude missing values: Listwise), rice yield was extremely significantly (P< 0.01) positively correlated with productive panicles number, total spikelets number, transplanting seedlings number, jointing stems number, dry matter accumulation during the heading to maturity stage, LAI, photosynthetic potential of jointing to heading, photosynthetic potential of heading to maturity, net assimilation rate during the heading to maturity stage, and NSCs. The productive panicles number was extremely positively (P< 0.01) correlated with total spikelets number, transplanting seedlings number, jointing stems number, heading stems number, dry matter accumulation during the heading to maturity stage, LAI, photosynthetic potential of jointing to heading, photosynthetic potential of heading to maturity stage, net assimilation rate during the heading to maturity stage, and NSCs, extremely negatively (P<0.01) correlated with the full grains number, seed setting rate, photosynthetic rate of flag leaf, and net assimilation of jointing to heading. Full grains number was extremely significantly (P<0.01) positively correlated with the seed setting rate, productive panicles rate, photosynthetic rate of flag leaf, extremely significantly (P<0.01) negatively correlated with jointing stems number, heading stems number, dry matter accumulation during the jointing to heading, LAI of jointing and maturity, indicating that single panicle traits must be combined overall material accumulation in the population to promote the transport of assimilates to the grains and achieve a good “sink.” In this study, the actual yield, productive panicles number, and full grains number per panicle were significantly (P<0.05) positively correlated with the NSC content, indicating that increasing the accumulation and transport of carbohydrates per unit area is beneficial for the synergistic improvement of rice yield via these two factors. In the present study, the WNDM resulted in greater productive panicles number and reciprocal second leaf photosynthetic rates, as well as increased material and NSC accumulation during the heading–maturity stage, resulting in greater yields than the other modes.

**Figure 9:**
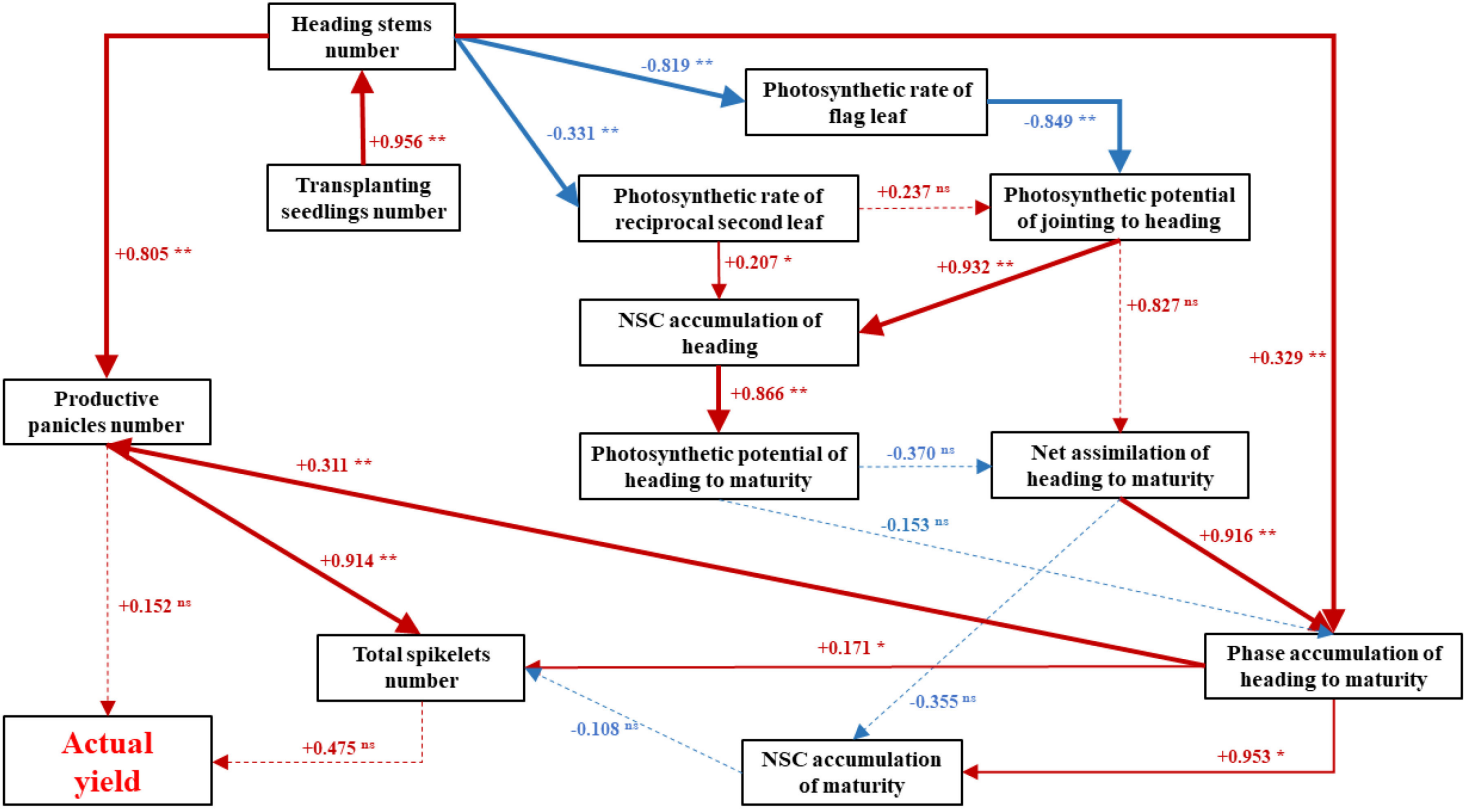
Interrelatedness map showing the potential mechanisms of row and hill spacing patterns to improve rice yield by regulating agronomic traits. A red arrow with a “+” indicates a positive effect; a blue arrow with a “−” indicates a negative impact. The solid line denotes that the impact is statistically significant, and the thicker the line, the greater the impact. The dotted line indicates that the impact is not statistically significant. * = significant at *p< 0.05*; ** = significant at *p< 0.01*.

Structural equation models (SEMs) were used to calculate the path coefficients between rice yield and agronomic traits (**Figure 9**). The actual yield was positive influenced by the productive panicles number (path coefficient 0.152) and total spikelet number (path coefficient 0.475). The productive panicles number was extremely significantly influenced by heading stems number (path coefficient 0.805) and phase accumulation of heading to maturity (path coefficient 0.311). The heading stems number was extremely significantly influenced by transplanting seedling number (path coefficient 0.956). The total spikelets number was extremely significantly influenced by productive panicles number (path coefficient 0.914), and significantly influenced by phase accumulation of heading to maturity (path coefficient 0.171). The phase accumulation of heading to maturity was extremely significantly influenced by net assimilation of heading to maturity (path coefficient 0.878), heading stems number (path coefficient 0.329) and influenced by photosynthetic potential of heading to maturity (path coefficient -0.153). This indicates that pursuing the individual photosynthetic parameters alone is not conducive to phase accumulation, and a balance between the quantity and quality of stems number should be sought. The indirect effect of row and hill spacing patterns on phase accumulation of heading to maturity was mainly positive attributed to its significant impact on photosynthetic potential of jointing to heading, NSC accumulation of heading and photosynthetic rate of reciprocal second leaf. Heading stems number had a direct extremely significantly negative effect on photosynthetic rate of flag leaf (path coefficient −0.819) and photosynthetic rate of reciprocal second leaf (path coefficient −0.331).

## 4 Discussion

Appropriately configuring rice row and hill spacing is an important means to establish a physiologically optimized population structure and improve effective access of crops to environmental resources. It can mitigate tradeoffs between individuals and populations and between the accumulation and transport of photosynthetic substances, achieve optimal spatial distributions, and increase yield (Zhu et al., 2012; Yang et al., 2024).

### 4.1 Relationship of row and hill spacing to rice yield and its components

Appropriate plant spacing can improve crop yield and optimize the structure of yield components, and these scholars have proposed corresponding technical measures (Peng et al., 2010; Wang et al., 2016). Rice yield can be increased by optimizing the cultivation mode, and this is also the most effective way to narrow the yield gap (Foley et al., 2011; Peng et al., 2015; Zhang et al., 2024). Li et al. (2015) reported that under high-density configurations with spacings of 15 cm × 30 cm and 20 cm × 25 cm, sufficient panicles can be formed, the canopy structure is optimized, and lodging risk can be reduced, providing guarantees of high yield and high quality. In their study on rice yield under different planting modes, He et al. (2023) reported that an excessive number of tillers in the population results in very limited individual space and intense competition for resources and hinders the formation of high yields. Lv et al. (2024) studied the cultivation of Zhuoliangyou 0985 under different fertilizers and planting densities and reported that the 20 cm × 20 cm densification method at a nitrogen fertilizer level of 180 kg/ha increased rice yield. Compared with the LFM, the densification modes resulted in an increase in yield. The WNDM (with a row spacing of 36 + 14 cm × 16 cm) increased the yield by 8.98% and 8.43% (**Figure 1**; **Table 1**), respectively, whereas the NDM (with a row spacing of 25 cm × 17 cm) increased the yield by 3.76% and 2.74%, respectively, relative to the LFM. This finding indicates that an increase in transplant density can be used as a measure to improve the yield of rice during large-scale mechanized production.

Previous studies have shown that increasing transplant density can increase the effective number of panicles, dry matter accumulation, and thousand-seed weight of a population, whereas under the same conditions, the productive panicle number and thousand-seed weight of rice gradually decrease (Song et al., 2024; Ye et al., 2024). In this study, the productive panicles number of significantly increased under the densification mode (**Figures 3**, **4**), whereas the number of grains per panicle and the seed setting rate decreased. The change in thousand seed weight was not significant (P>0.05), but the total spikelet number significantly (P<0.05) increased. This finding indicates that the densification mode can result in a greater storage capacity, meeting the accumulation requirements for grain materials. The total spikelet number was the highest under the NDM, but the actual yield under the NDM was lower than that under the WNDM, this disparity arose due to the NDM exhibited the lowest seed setting rate (**Table 1**). Although NDM achieved the highest productive panicle number (433.72/m²,441.70/m², **Table 1**), its yield remained 4.79% and 5.25% lower than WNDM. The excessive panicle density in NDM intensified intra-plant competition for photosynthetic assimilates (**Table 3**). Consequently, post-anthesis dry matter accumulation (**Table 2**) in NDM was 22.48% and 22.47% lower than WNDM of 26.36% and 25.53%, insufficient to support grain filling across all spikelets, restricting sucrose transport capacity. This is corroborated by the lower stem NSC (non-structural carbohydrate) translocation efficiency in NDM, which directly contributed to its lower full grains number. These findings highlight that simply increasing panicle density without optimizing canopy structure exacerbates source limitations and vascular constraints, ultimately capping yield potential. The NDM results in high storage sink capability, assimilates are not sufficiently transported to the grains, and the yield of this mode advantage cannot be fully realized. The WNDM optimizes the number of productive panicles and the number of full grains per panicle to improve rice yield.

### 4.2 Relationship of row and hill spacing to rice material accumulation and transport

Rice yield depends on the production capacity of the plants for photosynthetic substances and the transport and distribution of photosynthetic products and that the net accumulation of dry matter and its transport to grains in the middle and later stages of growth stage are particularly important for yield formation (Santiago-Arenas et al., 2019; Xu et al., 2019; Zhang et al., 2021). The variety selected in this study, Liaojing 419, is a variety that balances panicle and grain production. Ensuring the number of productive panicles and grains while maintaining a stable thousand seed weight and seed setting rate is the key to yield formation. In this study, the NDM resulted in the greatest number of stems and panicles during the jointing stage, heading stage, and maturity stage, but its productive panicle rate was not the highest (**Figure 3**). On the other hand, the WNDM optimized the productive panicle rate due to the relatively high number of stems during the heading stage, providing more productive panicles for the maturity stage and increasing yield. The lower planting density in LFM (18.52 hills/m²) reduced inter-plant competition for light, water, and nutrients during vegetative growth. Consequently, LFM plants allocated more biomass to structural growth, resulting in higher individual characters. LFM’s early individual vigor, its sparse population structure (LAI 6.22 and 5.96 at heading, **Table 3**) led to underutilized photosynthetic potential during grain filling. In contrast, WNDM’s optimized wide-narrow row configuration sustaining photosynthetic activity post-heading. This enabled WNDM to maintain a higher Net assimilation rate (1.78 and 1.71 g/m^2^/d, **Table 3**), ultimately surpassing LFM in total biomass through population-level efficiency rather than individual plant performance. These findings underscore a critical trade-off: while low-density systems (LFM) maximize early individual growth, high-density designs (WNDM) leverage spatial optimization to amplify collective productivity, particularly during reproductive stages when resource demands peak. Therefore, when the transplanting density is increased, attention should be given to the microenvironments of individuals and the creation of an effective population to achieve yield improvement.

Xu et al. (2007) reported that the planting mode employed can affect the aging rate of post-anthesis leaves, causing changes in ABA and ACC contents in grains. A high ABA/ACC ratio is beneficial for increasing the activity of enzymes in stem sheaths, promoting starch hydrolysis, increasing the soluble sugar content in stem sheaths, and promoting the transport of assimilates to grains. Compared with the other treatments, the WNDM significantly increased the accumulation of dry matter during the heading–maturity stage, resulting in a higher population growth rate, promoting the transport of photosynthetic substances to grains, and improving rice yield (**Figure 6**; **Table 2**). Moreover, the accumulation of NSCs in the stem sheath under the WNDM was the highest during the heading and maturity stages (**Figure 7**), with a relatively high transport rate and the highest sugar spikelet ratio, which allows for the “extraction” of substances from stem and leaves organs and promotes grain development during storage.

### 4.3 Relationship of rice row and hill spacing to leaf traits and photosynthetic characteristics

Morphological structure of rice leaves is an important indicator of plant type, and it is closely related to the light energy utilization efficiency of the plants (Yang et al., 1996). Achieving an appropriate spatial distribution of crops is not only conducive to building a reasonable canopy structure and resolving tradeoffs between individuals and populations, but it can also change the plant canopy structure, directly affecting the vertical distribution of light and temperature in the crop canopy and thereby indirectly affecting crop photosynthesis (Xiong et al., 2021, 2022). Deng et al. (2022) and Deng et al. (2023) reported that spatial distribution affects canopy structure and population photosynthetic efficiency, leading to changes in rice canopy microecology and causing changes in the rice grain-filling rate and ability in the later stages of growth. Xiong et al. (2022) reported that in the high-efficiency planting mode consisting of wide and narrow rows, the configuration mode involves “squeezing the middle row and empty side row” to form a ventilated and transparent “corridor” in the wide row while changing the “horizontal light” to “three-dimensional light,” fully utilizing the marginal advantages of the population, thereby improving the PAR interception rate of the middle and lower layers, especially the middle layer, and realizing the potential yield advantages of the variety. WNDM mode achieved a higher light penetration ratio in canopy due to northeast China’s longer photoperiod (14–16 h in heading-maturity stage, **Figure 1**) and lower solar zenith angles, which amplify wide row light diffusion benefits. WNDM principles (heterogeneous spacing) are universally relevant, optimal parameters must adapt to local conditions, in high-rainfall of grain filling stage, wide rows to 36 + 14 cm could mitigate humidity-driven disease pressure, this provides an environmental foundation for rice yield.

In this study, the productive panicles number was positively correlated with the net photosynthetic rate of the flag leaf, negatively correlated with the photosynthetic rate of the reciprocal second leaf, and significantly positively correlated with other agronomic traits. This finding indicates that the canopy of a single plant contributes differently to the formation of productive panicles and yield under different conditions. On the basis of the data (**Tables 2**, **4**), it can be determined that the WNDM, with a relatively high photosynthetic rate of the reciprocal second leaf, had relatively high yield, whereas the NDM, with the lowest photosynthetic rate of the reciprocal second leaf, had relatively high productive panicles, but its yield advantage was not obvious. This suggests that, compared with the NDM, the WNDM improves the top structure of the canopy for yield formation. In this study, the photosynthetic rate of the reciprocal second leaf under the WNDM was significantly greater than that under the other modes (**Table 3**), and the difference in the photosynthetic rate with respect to the flag leaf was the smallest, indicating that the improvement in photosynthesis in the upper and middle layers of the canopy caused by the narrow row spacing provides an environmental basis for yield improvement.

Canopy is a key determinant of light energy utilization in crops, and a reasonable canopy structure is beneficial for improving the photosynthetic performance of crop populations and unleashing the production potential of varieties. Therefore, adopting different spatial configurations and developing a reasonable canopy structure to improve population photosynthetic efficiency are highly important measures for increasing crop yield (Liu et al., 2022; Fu et al., 2011; Zhu et al., 2020). Rice is significantly more efficient at absorbing and utilizing light energy when it is planted in wide and narrow rows than in other planting modes, which may be related to the stability and regulatory capacity of the rice photosynthetic system (Liu et al., 2020; Dong et al., 2023). Wang (2017) suggested that the top leaves of the rice canopy are exposed to more solar radiation than other leaves area and have a higher transpiration rate. The content of cytokinins is higher in the top leaves of the canopy, which upregulates the expression of nitrogen export related genes (such as *OsAAP6*) in the aging leaves at the base, promoting the transport and distribution of nitrogen from aging leaves to the top leaves. In this study, the leaf area index under the densification mode was significantly greater than that under the LFM, providing a basis for the production of photosynthetic substances. The net assimilation rate during the heading–maturity stage under the WNDM was significantly greater than that under the other modes, promoting grain filling and yield formation.

### 4.4 Limitations

While this study demonstrates the agronomic benefits of wide narrow row densification mode (WNDM), several practical limitations must be acknowledged. First, WNDM requires specialized machinery and modified field management practices, which may increase initial investment costs for small-scale farmers. Second, the current findings are based on controlled field trials in mechanized production area of rice plain, the scalability of WNDM to diverse environments (e.g., hilly and mountainous regions) remains untested. Additionally, labor adjustments during mechanized operations (e.g., optimizing seedling density and machine settings) could pose challenges during large-scale adoption. To address these limitations, future studies should prioritize: Quantifying the cost-benefit ratio of WNDM adoption, including machinery depreciation, labor inputs, and yield gains under varying farm sizes, validating WNDM performance in contrasting environments.

## 5 Conclusions

In this study, an in-depth analysis of the impact of rice planting patterns on yield was conducted. Through field experiments with different mechanized transplanting modes, the significant effects of plant spacing configuration on rice yield, photosynthesis, and material transport characteristics were revealed. The wide and narrow row configuration of mechanized transplanting not only increased yield but also effectively optimized the spatial structure of the population, improved resource utilization efficiency, and increased production adaptability and promotion potential. This study provides theoretical and practical references for improving rice production efficiency and promoting high-quality mechanized processes in Northeast China, which is highly important for achieving sustainable development in modern agriculture.

## Data Availability

The original contributions presented in the study are included in the article/**Supplementary Material**. Further inquiries can be directed to the corresponding author.
