# Supplementary figures and images for: Optimization of row and hill spacing patterns improved rice population structure and increased rice yield
Source: Front Plant Sci. 2025 May 26;16:1570845. doi: 10.3389/fpls.2025.1570845 (PMC12146203; doi:10.3389/fpls.2025.1570845)

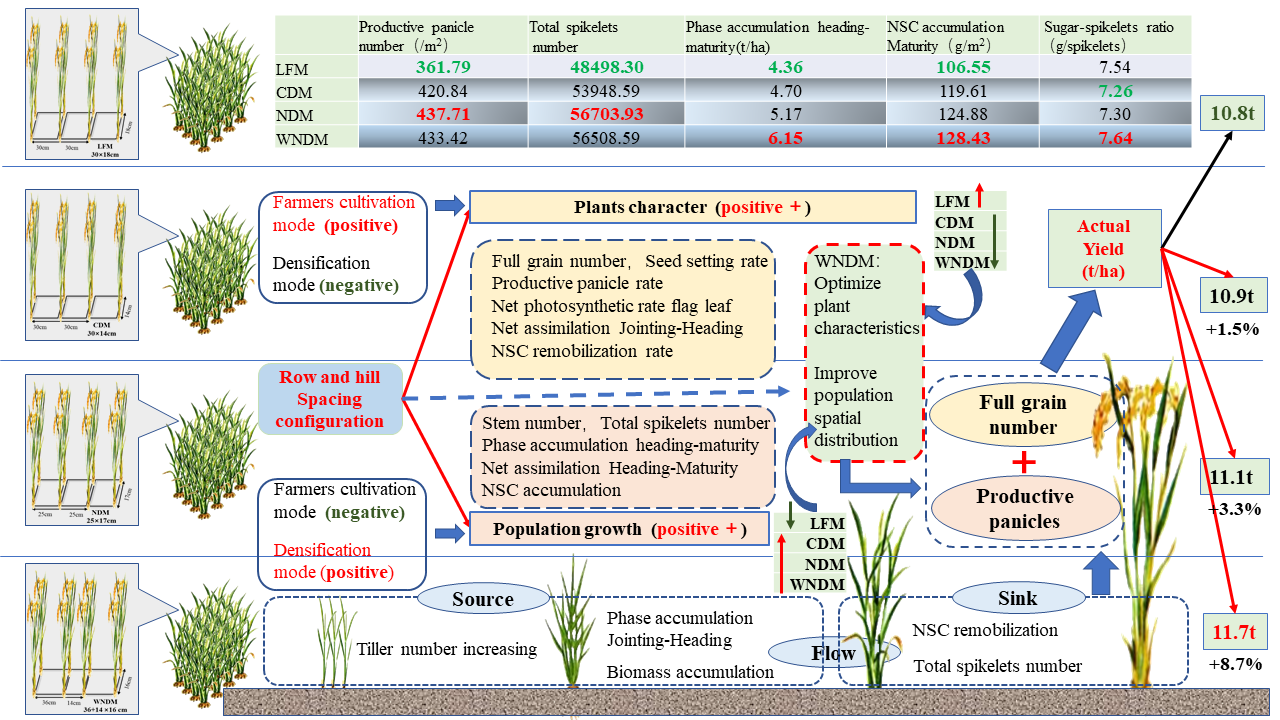

Supplement: Supplementary file 1 [file Image1.tif]
